# Epithelial Dysplasia in Oral Cavity

**Published:** 2014-09

**Authors:** Samaneh Shirani, Neda Kargahi, Sayed Mohammad Razavi, Solmaz Homayoni

**Affiliations:** 1 Department of Oral and Maxillofacial Pathology, Islamic Azad University of Khorasgun, Isfahan, Iran;; 2 Department of Oral and Maxillofacial Pathology, Isfahan University of Medical Sciences, Isfahan, Iran;; 3 Department of Oral Pathology, Isfahan University of Medical Sciences, Isfahan, Iran

**Keywords:** Dysplasia, Review, Iran

## Abstract

Among oral lesions, we encounter a series of malignant epithelial lesions that go through clinical and histopathologic processes in order to be diagnosed. Identifying these processes along with the etiology knowledge of these lesions is very important in prevention and early treatments. Dysplasia is the step preceding the formation of squamous cell carcinoma in lesions which have the potential to undergo dysplasia. Identification of etiological factors, clinical and histopathologic methods has been the topic of many articles. This article, reviews various articles presenting oral cavity dysplasia, new clinical methods of identifying lesions, and the immunohistochemical research which proposes various markers for providing more precise identification of such lesions. This article also briefly analyzes new treatment methods such as tissue engineering.

## Introduction

In oral mucosa we might encounter some epithelial or mesenchymal lesions. Based on epidemiologic research, the epithelial lesion could be the cause of most malignancies. Clinicians need to know the procedure of turning a malignant lesion to carcinoma.^1^^,^^2^ Awareness of epidemiology of oral cancers, especially squamous cell carcinoma, may provide effective and appropriate treatment plans, mortality reduction and enhanced life quality. Squamous Cell Carcinoma (SCC) is the highest prevalence lesion among other oral malignancies in epidemiologic studies.

Idris et al. in Sudan reported 66.5% prevalence of SCC among malignant lesions.^3^ In 2007, Razavi et al. claimed that epithelial malignancies were the most prevalent lesions (63%) and reported 54.5% SCC prevalence in Isfahan.^4^ As some scientists believe that early diagnoses of lesions are important in both prevention and therapeutic procedures of oral cancers, many clinical, histological and cytological studies have been carried out. While few focused on clinical evaluation of different lesions diagnoses methods, few others recommend modern cytological method used in genecology. Despite the availability of such modern methods, pathologists claim that microscopic view of biopsies makes higher specificity and sensitivity in diagnosis.^5^^-^^7^

*At first, Definition of Few Terms*

*Precancerous Lesion;* refers to a tissue with benign morphological change having high potential to turn into malignance.

*Malignant Transformation Potential; *refers to occurrence of malignancy or precancerous either in primary or final stage. Dysplastic changes are one of the sings of premalignant lesions which can be observed in histopathologic view especially in epithelial. Although recent development in medical science^8^^-^^10^ recommend conservative methods, additional research is required for further evaluation. The dysplastic epithelial lesions have the clinical characteristics of a premalignant lesion similar to carcinoma. Recognition of such change is vital in preventing carcinoma changes.^10^ Premalignant lesions such as leukoplakia, erythroplacia, smokeless tobacco keratosis, oral sub mucosal fibrosis, Lichen Planus, Condylom Acominatum, Inverted Schneiderian  Papilloma Actinic Keratosis are briefly discussed below.^7^^,^^10^^,^^11^

*Leukoplakia: *A white plaque or patch without any clinical or pathological similarity to other lesions. This term is entirely clinical without including any histopathologic tissue change. Dysplastic epithelium or invasive carcinoma is only observed in 5 to 25 % of sample biopsies. The etiologies of leukoplakia are commonly tobacco, alcohol, sanguinaria, ultraviolet radiation, trauma and microorganism like treponema palladium, candida albicans and papilloma virus. This lesion is mainly seen in people less than forty years old and prevalent in males. Although vermilion, buccal mucosa and gingiva are the most common location of leukoplakia; lesion positioned on lips, tongue and oral floor have 90% likelihood to represent dysplastic or carcinoma changes.

The clinical process of leukoplakias is described below:

Thin leukoplakia appears in a white-gray or gray plaque and may have fissures and wrinkle appearance.

Thick leukoplakia is a white plaque with obvious thickness, leathery palpation and numerous deepen fissures.

Nodular/glandular leukoplakia is more sever with more surface irregularities.

Verucous/verrociform leukoplakia has sharp or blunt projections.

Proliferative verrucous leukoplakia is a high risk type with multiple keratotic plaque and roughened surface projections.

**Figure 1:**
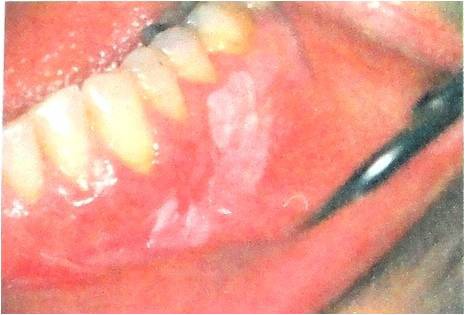
Leukoplakia is a white plaque or patch without any clinical or pathological similarity to other lesions.

Histopathologic feature of leukoplakia is a thickened keratin layer of epithelium (hyperkeratosis) with or without thickened spinouts layer (acanthosis). Some demonstrate epithelial atrophy. Different inflammatory cells are observed in connective tissue. Most lesions demonstrate no dysplasia on biopsy except for 5-25%, if all oral sites explored. These dysplastic, if exist, typically begins in the basilar and parabasilar of the epithelium^7^^,^^10^^,^^12^^-^^15^, as showing in figure 1.

**Figure 2:**
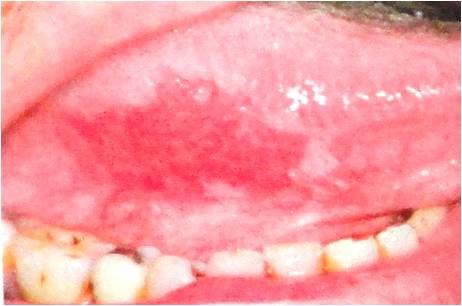
Erythroplakia is defined as a red patch that cannot be diagnosed as any other lesion clinically and pathologically.

*Erythroplakia: *This lesion is defined as a red patch that cannot be clinically and pathologically diagnosed as any other lesion. Obvious dysplastic changes and carcinoma in situ or invasive squamous cell carcinoma are present almost in all true lesions. Etiology of erythroplakia is unknown but assumed to be similar to invasive squamous cell carcinoma etiologies. Although the prevalence of erythroplakia is rare, the occurrence of sever dysplasia during biopsy or further malignant lesions is likely. The prevalence of erythroplakia is mostly in middle aged adults without specific gender predilection. Floor of the mouth, tongue and soft palate are the common site of involvement in which the lesion exhibits a well-demarcated erythematous macule with a soft and velvety texture. Histopathologic feature reveals sever epithelial dysplasia, carcinoma in situ and invasive squamous cell carcinoma in 90% of cases. There is an atrophic epithelium with lack of keratin production specially when combined with epithelial thinness allowing the underlying microvascluar to present itself with red appearance. As shown in figure 2, the connective tissue often demonstrates chronic inflammation.^7^^,^^10^^,^^16^^-^^19^

**Figure 3:**
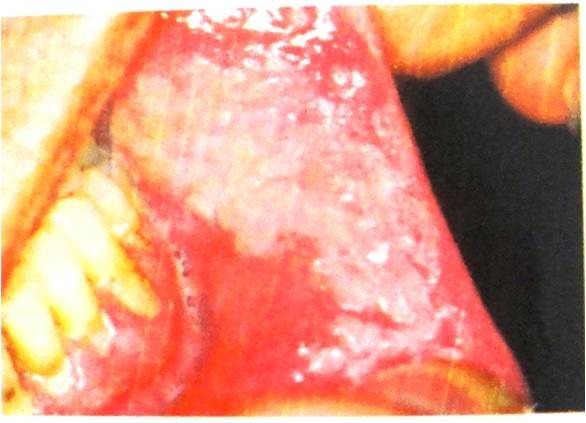
Smookless tobacco patch appears as a characteristic thin white- gray or white plaque on mucosa in direct contact with snuff or chewing tobacco.

*Smookeless Tobacco: *The lesion appears with a characteristic of thin white- gray or white plaque on mucosa in direct contact with snuff or chewing tobacco with ill-defined border and little erythema. If occurs, smokeless tobacco keratosis takes up to five years to emerge. It becomes permanent unless daily contact with tobacco is avoided. Some white lesions become thicker with leather or nodular appearance. In histopathology view, hyperkeratinized and acanthotic squamous epithelium with or without cellular vacuolization or edema of Glycogen-rich cells are seen. Parakeratin chevron can be seen as pointed projections above or within superficial epithelial cells. Dysplasia, if exist, is mild in this lesion (figure 3).^7^^,^^10^^,^^20^^-^^23^

**Figure 4:**
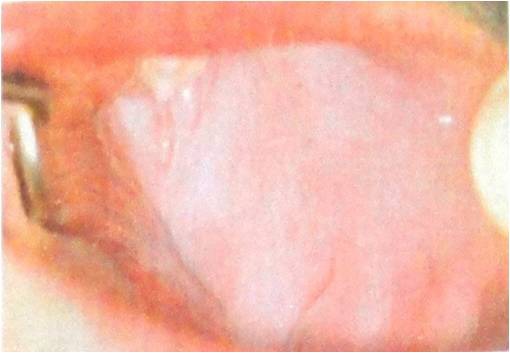
Clinical finding of submucous fibrousis special characteristic is a mucosal rigidity of varied intensity.

*Submucous Fibrosis: *This is a chronic progressive scarring lesion with high-risk precancerous condition which has been linked to placement of “paan” in the mouth. Its special characteristic is a mucosal rigidity of varied intensity caused by fibroblastic hyperplasia and modification of superficial connective tissue. Nutritional deficiency and genetic factors increases the risk of fibrosis and tobacco contact is considered as the major cause of epithelial alteration and carcinogenesis. Disruption of the hemostatic equilibrium between synthesis and degeneration which is caused by areca is its pathogenesis. The copper ”ion” in areca “nuts” increases the activity of lysyl oxidase leading to unregulated collagen production. The lesion mostly involves young adults and the most affected sites are buccal mucosa, retromolar area and soft palate. Deposition of dens and hypervascular collagen in connective tissue with variable numbers of chronic inflammatory cells are seen in its histopathologic features. Epithelial dysplasia has been noted in 10 to 15% of biopsies (figure 4).^7^^,^^10^^,^^24^^-^^28^**

**Figure 5:**
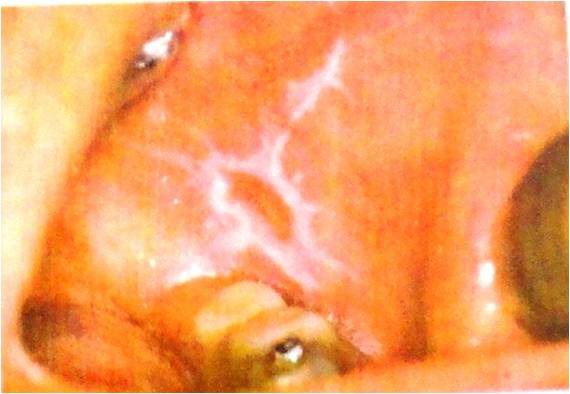
Clinical finding of lichen planus reticular type is more common and involves the posterior buccal mucosa bilaterally with interlacing white lines appearance.

*Lichen Planus: *A chronic dermatologic disease often affects oral mucosa without any specific etiology including drugs, C hepatitis and nervousness. It is common in middle aged adults and associated with immunological disorders. Reticular type is more common than the erosive form and involves the posterior buccal mucosa bilaterally with interlacing white lines appearance. Other sites that could be affected are; lateral and dorsal tongue, the gingiva, the palate and the vermilion border. The clinical view in the erosive type is an atrophic, erythematous with central ulceration and fine peripheral, white radiating stera areas. Histopathologic features are diverse degrees of orthokeratosis and parakeratosis besides thickness of spinous layer. Rete ridges become hyperplastic and saw-toothed shape pattern. Degeneration of basal cells alongside with an intense, band like infiltrated T lymphocytes is evident. Malignant changes of lichen plans are controversial but if dysplastic changes occurs, it would be mild (figure 5).^7^^,^^10^^,^^29^^-^^31^

**Figure 6:**
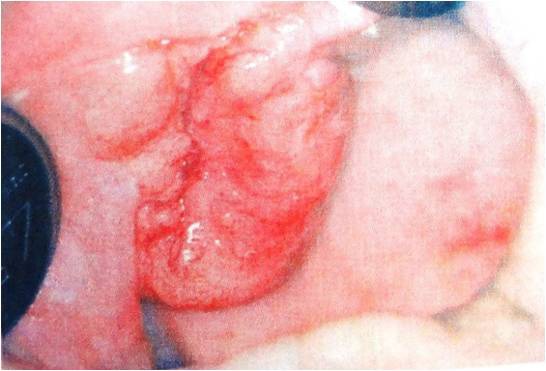
Clinical finding of Inverted Schneiderian papilloma appearance is a soft, pink and polypoid growth.

*Inverted Schneiderian Papilloma: *It is the most common sinonasal papilloma that mainly observed in middle aged adults. Unilateral nasal obstruction alongside with pain, epistaxis and purulent discharge are the symptoms. Its appearance is a soft, pink or tan, polypoid or nodular growth with histologic features of proliferated squamous cell into the submucosal stroma. The basement membrane remains intact and diverse degrees of dysplastic changes are evident. Obvious hyperplasia and hyperkeratosis in basal layer and high mitotic index have made poor prognosis (figure 6).^7^^,^^10^^,^^32^^-^^36^

**Figure 7:**
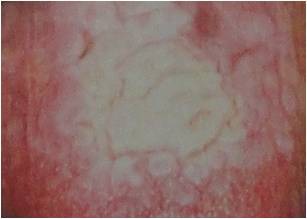
Clinical finding of actinic keratosis are atrophic lower lip distinguished by a smooth surface and blotchy pale areas.

*Actinic Keratosis: *The lesion is a common cutaneous premalignant caused by cumulative ultraviolet radiation of the sun on the exposed skin. Actinic cheilosis is the type in which it affects the lower lip vermilion and is common in white male adults. This lesion is also called farmer’s lip or sailor’s lip. Compromised immunity and long exposure to the sun are its most etiologies. Clinical features are atrophic lower lip distinguished by a smooth surface and blotchy pale areas. In progressed lesions; rough, scaly areas are developed in drier portion of the vermilion which is similar to leukoplakia lesions. Atrophic stratified squamous epithelium with remarkable keratin productions and varying degrees of dysplasia are seen in histopathologic features. Additionally, a mild chronic inflammatory cell and solar elastosis are present in underlying connective tissue (figure 7).^7^^,^^10^^,^^37^^-^^40^

## What Are the Clinical Features of Dysplastic Lesion and Whether Sufficient to Diagnose a Dysplastic Lesion or Biopsy Is Necessary?

Observing a white keratotic lesion with unknown or similar to malignancies etiology, suggest the possibility of dysplastic changes. In 1876, a Hungarian dermatologist detected leukoplakia.^41^ Schwimmer spotted that 80% of leukoplakia lesion are premalignant lesion and the possibility of malignancy increased by 3% per year in adults older than 35-year-old.^42^ In further studies, it was concluded that every white keratotic lesion does not necessarily have malignancy potential due to their response to mucosal preservation functions. In a workshop,*^7^* pathologists presented the following keratotic lesion as having high potential malignant and dysplastic changes; lichen plans, smokeless tobacco, alveolar keratosis and other similar leukoplakia lesions. The likelihood of dysplastic changes is higher in thicker and more granular lesions. Furthermore, the existence of red spots in white lesions determines higher incidence of malignancy. It is claimed that multi-focal leukoplakia have high potential of malignancy. Other mucosal lesions including red lesion like leukoplakia is suggested to have 5 to 25% of dysplasia. Similar probability for the case of erythroplakia is 90% (table 1).^10^

**Table 1:** Comparing the possibility of dysplastic changes in oral lesions is the likelihood of dysplasia in dysplastic lesions

Proliferative verrucous leukoplakia	******
Nicotine palatinus in reverse smoking	*****
Erythroplakia	*****
Oral sub mucus fibrous with leukoplakia	*****
Granular leukoplakia	****
Laryngeal keratosis	***
Actinic cheilitis	***
Syphilitic glossitis with dorsal leukoplakia	***
Smooth, thick leukoplakia	**
Smokeless tobacco keratosis	**
Plammer Vinson disease	*
Lichen planus ,erosive form	*
Smooth, Thin leukoplakia	+/-
Dyskeratosis congenital	?
Lupus erythematous	?
Epidermolysis bullosa	?
Clarke-Howel-Evans syndrome	?

^ ^

The chances of malignancy in mild or moderate dysplastic lesions are 4 to 11% and 2 to 35% for severe dysplastic changes. Also it has been surveyed that a premalignant lesion takes approximately up to 3 years to turn into an oral cancer. Several studies evaluated the possibility and capability of malignancy changes but could not be identified nor proved with certainty.^43^^-^^46^ Defining the impact of molecular factors would be helpful in determining and discovering therapeutic methods. Many scientists have put a lot of effort in determining the histopathologic etiology of these changes. The results have shown that some markers and histopathologic elements are relevant. A study by Razavi et al. demonstrated that vascularization with VEGF has paramount role in dysplasia progression and carcinomas from a normal mucosa.^47^ Lectine is a membrane protein marker which attaches to the membrane carbohydrate and have function in cell membrane. It has been proved that lectine has a role in oral, breast and brain cancers. Mutation in the gene of lectine, alters cell membranes and leads to metastatic tumoral cells.^48^^-^^51^ A study by Silverman concluded that 36% of leukoplakia ends with malignancy. 7-50% of sever dysplastic lesions, 3-30% moderate dysplastic lesions and lesser than 5% of mild dysplastic lesions are capable of turning into malignancies.^52^^-^^55^ Many pathologists believe that dysplastic changes are temporary as an incipient stage of turning to malignancy and a mild dysplasia might lead to sever dysplasia.^56^

## What Is Histopathologic Feature of a Dysplastic Lesion as the Most Important Factor in Diagnosis and Prognosis?

Dysplastic changes, if occurs, embark upon basal and parabasal epithelium. The more dysplastic changes occur, the more unusual epithelium spread across whole epithelium. The words “mild”, “moderate” and “sever” are used to describe the severity of dysplasia.^10^

**Figure 8:**
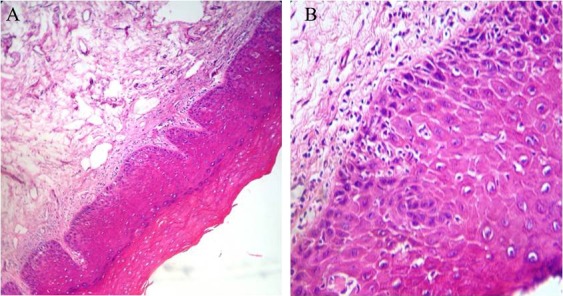
Histopathology finding of mild dysplasia describes changes from basal or parabasal layer (A: magnification ×100, B: magnification ×400).

Mild dysplasia refers to changes limited to basal or parabasal layer (figure 8).

**Figure 9:**
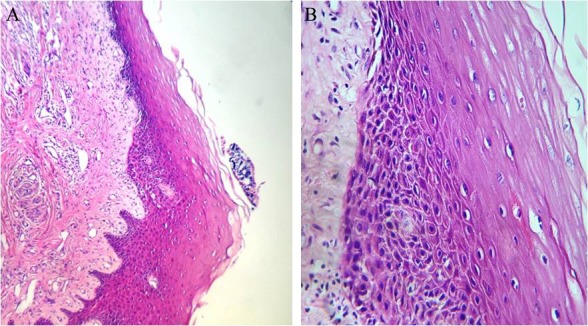
Histopathology finding of moderate dysplasia describes changes from basal layer to middle of granular layer (A: magnification ×100, B: magnification ×400).

Moderate dysplasia involves basal layer to middle of granular layer (figure 9).

**Figure 10:**
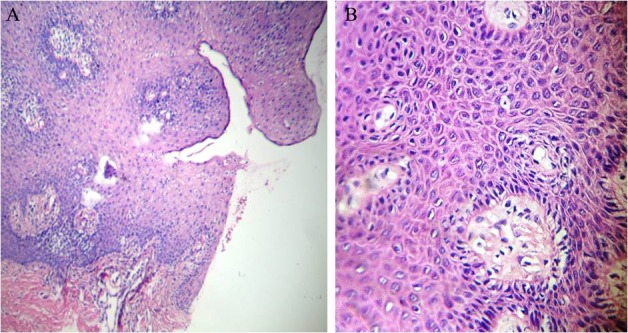
Histopathology finding of sever dysplasia, describes changes from basal layer to upper and middle layer of epithelium (A: magnification ×100, B: magnification ×400).

Sever dysplasia describes changes from basal layer to upper and middle layer of epithelium (figure 10).

**Figure 11:**
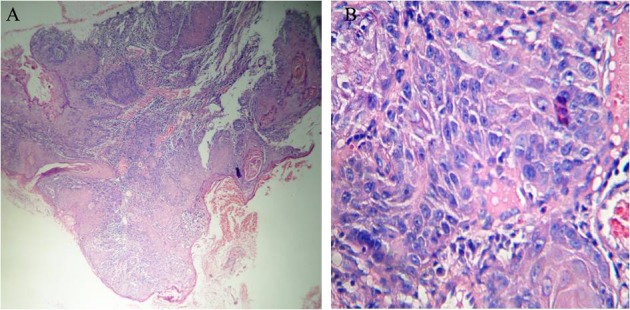
Histopathology finding of carcinom in situ is defined as dysplasia involved basal layer to surface of the mucosa  (A: magnification ×100, B: magnification ×400).

Carcinoma in situ is defined as dysplasia involved basal layer to surface of the mucosa which can spread through one salivary gland’s duct specially when located in oral floor. The point in carcinoma in situ is that the basal layer is intact and healthy (figure 11).^57^^,^^58^^,^^59^

## So What Are These Dysplastic Changes?

They are categorized into two common types:

*Cellular Changes*

Specific alterations of individual epithelial cells are important in the determination of epithelial dysplasia.^57^^-^^59^ Cells and nuclei take on more primitive appearance, similar to those of basal cells with enlarged nuclei (nuclear hyperplasia), dark-staining nuclei (hyperchromatism), often enlarged eosinophilic nucleoli (prominent nucleoli) and with increased nuclear-to-cytoplasmic ratio.^57^^,^^58^^,^^5^ These cells also show increased cellular density. Such changes are not exclusive to carcinogenesis, as they may be seen in reactive epithelium or epithelium influenced by a variety of systemic alterations. Flow cytometry and image cytometry significantly adds to the pathologist’s ability to assess nuclear changes associated with eventual cancer development.^57^^-^^59^

There is often an increase in mitotic activity in dysplastic epithelium but this is also seen in many reactive lesions .Multipular mitotic figures (abnormal mitoses) may also be defined in unusual location of other epithelium layers.^57^^-^^59^

*Tissue Changes and Appearances*

-Most oral precancerous lesions show hyperkeratosis and acanthosis, dysplastic lesions may be atrophic as well as acanthotic^57^^,^^58^^,^^59^
-Changes in maturity from basal cell layer to squamous cells, morphological alteration of dysplastic epithelium are loss of stratification (loss of polarization).^57^^-^^59^
-Basal cell hyperplasia, as discussed above, is of major importance to diagnosis as well as to the grading of dysplasia.^57^^-^^59^
-Carcinoma in situ
-Congestion of cells
-This dyskeratosis may be represented by individually keratinized cells or by tight concentric rings of flattened keratinocytes (epithelial pearls*)*.^57^^-^^59^
-Nodular, bulbous or tear like rete ridge and extremely elongated rete processes (drop-shaped rete processes), are of concern regardless of their size, especially if secondary projections or nodules are seen to arise from the basal layer and branch at indifferent angles into the lamina propria and connective tissue papillae.^57^^-^^59^

*Systems to Categorize Dysplastic Lesions*

Four systems for categorization of dysplastic lesions are invented:

*Oral Intraepithelial Neoplasm (OIN)* (table 2)

**Table 2:** OIN system

**OIN system**	**No dysplasia**
n/a	Mild dysplasia
OIN 1	Moderate dysplasia
OIN 2	Sever dysplasia
OIN 3	Carcinoma in situ
OIN 4	No dysplasia

*Classic Laryngeal System* (table 3)

**Table 3:** Classic laryngeal system

***Classic laryngeal system***	
Grade 1	Laryngeal keratosis
Grade 2	Keratosis with dysplasia
Grade 3	Carcinoma in situ

*Laryngeal Keratosis.…ljubljana system* (table 4)

**Table 4:** Ljubijana system

**Ljubljana system**	
Grade 1	Simple hyperplasia
Grade 2	Basal/parabasal hyperplasia
Grade 3	Atypical hyperplasia
Grade 4	Carcinoma in situ

*Classic oral system (WHO 2005) *(table 5)*.*^60^

**Table 5:** Classic oral system

**Classic oral system (WHO 2005) **
No dysplasia
Mild dysplasia
Moderate dysplasia
Sever dysplasia
Carcinoma in situ

## Is there any special method to diagnose a dysplastic change clinically?

Several researches with certain recommendations and methods have been carried out. However, their specificity and sensitivity is not as reliable as biopsy and histologic method. Some of those techniques are:

*Brush biopsy:* this method is based on Pap smear test, alternatively known as Oral CDx test. If possible, a brush is used with its bristle to smear lesion and its basal layer. The sample is transferred onto slide and dyed similar to Pap method. Possible malignancy is established if one of the following indications is observed; polymorphism enlarged nulears, dark and enlarged nucleus.^61^^-^^64^

*Liquid based cytology:* is apparently based on brush biopsy with more preserving cells and morphologies. In this technique the sedimentation procedure is used and inflammatory and blood cells are removed. The specificity and sensitivity of this test is higher with lesser false +/- results that permits immunohistological and molecular surveys. This technique is applicable when biopsy is doubtful (i.e. used for borderline lesions).^65^^-^^68^

*Toluidine blue:* rinsed mucosa is exposed to acetic acid followed by Toluidine blue dyes cell’s DNA which makes observing dysplastic changes possible. In this test more false+results might be provided due to destroyed cells by trauma or inflammation. Indication of this technique is carcinoma in situ, erythroplakia and finding locations for biopsy.^69^^-^^72^

*Vizilite technique:* suspicious mucosa is evaluated by blue light which is used in dentistry or chemical radiating tubes in a dark room after acid acetic exposure. The unusual mucosa would appear as dark zones. The weakness of this test is its false+results and exaggerating dysplasia in lukoedema and smokeless tobacco lesions.^7^^,^^73^^-^^75^

*Oral auto fluorescence**:*** Fluorescent light is used to diagnose endangered location in lesions and is appropriate for “keratotik” and vascular lesions. Fluorescent light with wavelength of 400-460nm in a dark room can localize danger zones as “blue”, “green and black” or “black and black” appearances. This might be due to decrease FAD and NADH activities which can absorb fluorescent light. For example, in leukoplakia with dysplasia, the number of black spots is 3-4 but in the same lesion with no dysplasia there would be 0-1. The weakness of this test, as in the previous method, is its low specificity and sensitivity.^7^^,^^76^^-^^78^

Immunohistologic technique and using mononuclear antibodies against specific markers of dysplastic cells can provide higher reliability. The percentage of positive results determines the type and severity of dysplasia. The recommended markers are: DOK,^79^ Rab11a,^80^ P53,^81^ POE9n,^82^ Bcl2,^83^ and CD44.^84^

## How Can Progress of A Dysplastic Lesion to Malignancy Be Prevented After Diagnose? Is Treatment Necessary or Not?

This would be different and depends on the lesion and the treatment plan. Eliminating habituated condition which is the cause of the problem is appropriate but nowadays, medical science recommends using stem cell treatment. In a relevant study, the number of mild, moderate and sever dysplastic samples treated by stem cells are reported with convincing results. The mucosa has gained the ability of producing normal keratins with maturity and function. Relying on the new method, diagnosing and treating dysplastic lesions is promising even in high possibility of malignancy returnings.^85^^-^^87^

*Tissue engineered oral mucosa: *Tissue engineering is defined as “understanding the principles of tissue growth and applying this to produce functional replacement tissue for clinical use^88^ Tissue engineered in oral mucosa can replace soft tissue defects in the oral cavity.^89^ These defects can be divided into two major categories; tooth-related defects (gingival recessions) and non-tooth-related defects (defected with truma, chronic infection or defects caused by oral cancers).^90^ Common approach for replacing damaged oral mucosa is the use of grafts and cultured epithelial cells.^89^ Tissue engineering in oral mucosa comprises of two techniques; Partial-thickness engineered oral mucosa and full-thickness tissue engineered oral mucosa. Partial-thickness technique allows production of epithelial cells for replacing dysplastic oral mucosa.^89^ This technique uses one type of cell layer which can be in monolayer or multilayer. Monolayer epithelial makes use of the response to stimuli such as mechanical stress, growth factor addition and radiation damage.^91^ These multilayer epithelial cells show signs of differentiation such as the formation of a basement membrane and keratinization.^92^ With the advancement of tissue engineering as an alternative approach, full-thickness tissue-engineered oral mucosa was developed.^92^ This technique is a better simulation of the in vivo situation as they take the anatomical structure of native oral mucosa into account. The main goal of the full-thickness technique is to resemble normal oral mucosa. To obtain best results, the subtype and origin of the fibroblasts and keratinocytes (used in oral mucosa tissue engineering) are important factors. Fibroblasts are usually taken from the dermis of skin or oral mucosa.^92^ Keratinocyte can be isolated from different areas of the oral cavity (the palate or gingiva).^92^ Time is an important parameter when using fibroblasts and keratinocytes, since the functioning of these cells decreases with time.^92^ Transplanted cells should adapt to their new environment and function correctly.^92^ There is a risk of losing transplanted tissue if cells do not adapt properly. This adaptation goes more smoothly when the donor tissue cells resemble the cells of the native tissue.^92^

## Conclusion

All investigators generally accept the fact that most carcinoma cases in oral cavity, display considerable change before reaching such state. Thus, dysplastic lesions with microscopic characteristic features have a high risk potential for transformation to malignancy. Therefore, appropriate diagnostic procedures (i.e. biopsy) for every suspicious dysplastic lesions in oral cavity should be considered.
